# Causal determinants of complex PTSD in Syrian refugee children living in informal settlements in Lebanon

**DOI:** 10.1192/j.eurpsy.2022.214

**Published:** 2022-09-01

**Authors:** C. Biazoli, M. Pluess

**Affiliations:** Queen Mary University of London, Experimental And Biological Psychology, London, United Kingdom

**Keywords:** Social Determinants, causal inference, PTSD

## Abstract

**Introduction:**

Displaced refugee children with a history of war exposure are at risk of developing complex and severe forms of post-traumatic stress disorder (PTSD).

**Objectives:**

Search for the most relevant causal predictors of complex PTSD in a prospective cohort of Syrian refugee children living in informal settlements in Lebanon (N=1007).

**Methods:**

A latent class unsupervised analysis was carried out to determine clusters with complex PTSD presentation at the follow-up assessment. A new exploratory causal discovering modelling approach was applied using 97 multilevel psychosocial variables as predictors (Biazoli et al., 2021). Associations between discovered candidate causal factors assessed at baseline with a presumed diagnosis of complex PTSD one year later were calculated using a multiple logistic regression model.

**Results:**

Several putative causal factors emerged: perceived social coherence of the neighbourhood (Positive Predictive Value increase: 1.22); impulsivity (1.25), self-efficacy (1.23) and depressive symptoms (1.15) at the parental level; positive home experiences (1.16) at the family level; and child-level factors such as being forced to work (1.22), being a victim of verbal or physical bullying (1.19), loneliness (1.17) and well-being (1.18). In further confirmatory multiple logistic regression analysis and after correction for multiple comparisons, verbal or physical bullying victimization (p=.005) and caregiver depressive symptoms (p=.0004) at baseline were associated with complex PTSD presentations one year later.

**Conclusions:**

Our results support the need for a multi-level psychosocial care model to prevent psychological distress and promote mental health in refugee children. Specifically, our results suggest that programs tackling caregiver’s mental health and children’s exposure to violence might effectively prevent complex PTSD.

**Disclosure:**

No significant relationships.

